# Evaluating the impact of an injury prevention measure regarding different sociodemographic factors

**DOI:** 10.5249/jivr.v10i1.952

**Published:** 2018-01

**Authors:** Thomas Brockamp, Paola Koenen, Manuel Mutschler, Michael Köhler, Bertil Bouillon, Uli Schmucker, Michael Caspers, . Working Group Injury Prevention of the German Trauma Society(DGU)

**Affiliations:** ^*a*^Department of Traumatology and Orthopedic Surgery, Cologne-Merheim Medical Center (CMMC), University of Witten/ Herdecke, Ostmerheimer Str. 200, 51109 Cologne, Germany.; ^*b*^Working Group of Injury Prevention of the German Trauma Society, The German Trauma Society, Straße des 17. Juni 106-108, 10623 Berlin, Germany.; ^*c*^Steinbeis Transfer Center for Research in Intervention and Evaluation, Wieksweg 48,33378 Rheda-Wiedenbrueck, Germany.; ^*d*^Department for Trauma Surgery, University Hospital Regensburg, Franz-Josef-Strauss-Allee 11, 93042 Regensburg, Germany.; ^*e*^AUC - Academy for Trauma Surgery, Wilhelm-Hale-Str. 46b, 80639 München, Germany.

**Keywords:** Prevention, P.A.R.T.Y program, Trauma, Youth

## Abstract

**Background:**

Road traffic collisions (RTC) remain a major problem especially among young road users. Injury prevention measures and licensing systems have increasingly been developed to counteract some of the negative effects of RTCs in youth. The Prevent Alcohol and Risk Related Trauma in Youth (P.A.R.T.Y.) program is an injury prevention program that promotes prevention through reality education. In this study, the impact of the program on different sociodemographic subgroups of school students was analyzed. The aim was to find out which subgroups were influenced the most and how improvements to the program can be made.

**Methods:**

Evaluation was performed in a pre-post-intervention setting by means of a standardized questionnaire. The questionnaire contained three different sections with a total of 22 questions to identify students' responses regarding risk-behavior and risk-assessment. Evaluation was done at two different points on the same day (pre- and post- intervention). Data were analyzed with a focus on gender, age, residential area and level of education. Cronbach's alpha was used to check all questions for reliability. Data were analyzed using the t-test and the Wilcoxon signed-rank test with significance defined as p less than 0.05.

**Results:**

The study sample contains 193 students (range 14-17 years of age, 44% male). Female students show better results regarding risk-behavior and risk-awareness. The same applies to students of a higher educational level. And students ≥ 16 years showed significantly better results in all three sections compared to younger students.

**Conclusions:**

Morbidity and mortality due to RTCs is a major problem in the group of young road users. Especially male road users between 14 and 17 years of age with a low educational level are at high risk to sustain road traffic injuries. Our results show that the P.A.R.T.Y. program has a stronger effect on young female students. Additionally, a significant effect was measured on students ≥ 16 years of age and on students with a higher educational level. Prevention measures need to be evaluated and further improved particularly in order to address the high-risk group of young, male road users with a lower educational status.

## Introduction

Young road users continue to be the most vulnerable group with a high incidence of mortality and morbidity in road traffic collisions (RTC).^1-3^ Although the child injury death rate is much lower among children from developed countries, injuries are still a major cause of death, accounting for about 40% of all child deaths.^4,5^ Road traffic injuries alone are the leading cause of death among 15 to 19-year-olds and the second leading cause among 10 to 14-year-olds. ^6,7^ In most regions of the world the problem of road traffic injuries is still increasing. However, RTCs not only affect the current low- and middle-income countries but also the industrialized regions in Europe and North America. ^3^ Each year, nearly 30,700 people die due to RTCs in Europe and approximately every two hours a person dies during RTCs in Germany. ^8^ The causes are largely known. Some of the reasons for most injuries are speeding, alcohol abuse and overestimation. ^9,6,10^ But lack of experience, life-style, and group pressure also figure prominently . ^11,12^

**Figure 1:**
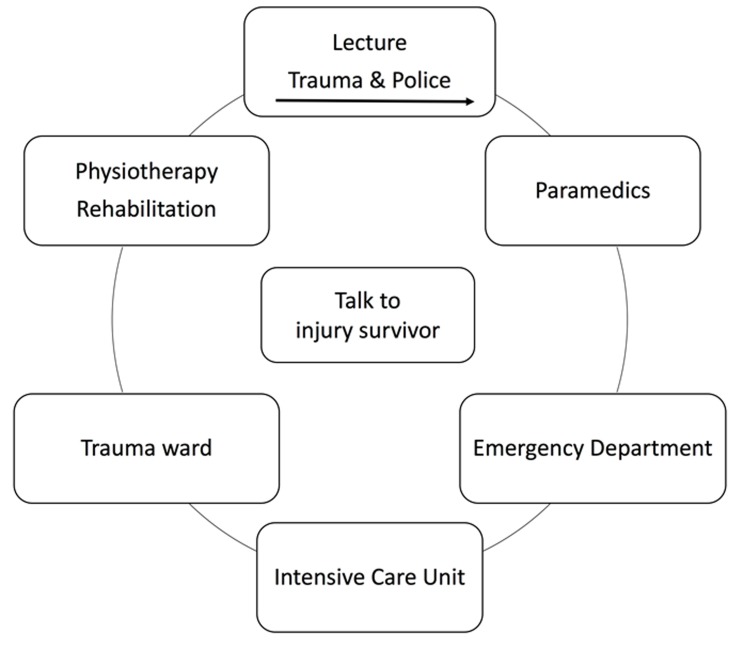
Structure of the P.A.R.T.Y. prevention program. Students start at the lecture room and finish the day with a talk to an injury survivor.

Injury prevention programs and licensing systems have increasingly been developed to counteract some of the negative effects of RTCs in youth. A lot of injury prevention strategies and methods have been set up to enlighten young road users about the consequences of trauma. Especially educational measures, community based measures as well as legislative measures were set up to reduce injuries in the young population. ^13,14^ Educational measures are known to reduce injury rates in the short term. ^13,14^ The P.A.R.T.Y. program (Prevent Alcohol and Risk Related Trauma in Youth) is an educational measure with a focus on young road users. It is a 1-day in-hospital injury awareness and prevention program for youth aged 14 years and older. The program provides information about trauma and its consequences and it may enable students to recognize potential injury-producing situations, make prevention-oriented choices and adopt behaviors that minimize unnecessary risk. Students spend about six hours of a single day in a trauma unit. The academic and nursing staffs of these trauma centers are specially trained in teaching the participants. The program starts with an interactive presentation held by a trauma surgeon who explains trauma and the way a severely injured patient is rescued and treated. Next, a session is presented by a local police officer, outlining risk-taking behaviors and possible consequences of bad choices, e.g. drug or alcohol use, no helmet use. Each presentation and discussion lasts about 20-30 minutes. After a break, students are divided into small groups and begin tours of the paramedic services, Emergency Department (ED), Intensive Care Unit (ICU), trauma ward and physiotherapy unit. The students are encouraged to touch and feel real equipment at mock bed spaces and to ask questions during the visit to the intensive care unit and trauma wards. The students are given the opportunity to meet and talk to injured survivors about their injuries and the choices that led to the experience of trauma. ^15^(Figure 1)

The program is a component of the growing community effort to reduce traumatic intentional and unintentional injury and death in youth, resulting from alcohol, drug, and risk-related crashes and incidents.^16,17^ There is evidence that this program can positively influence the behavior of young road users to reduce road traffic collisions and the severity of injuries.^18^ Banfield and colleagues published a 10-year retrospective analysis of the effectiveness of the program. P.A.R.T.Y. participants were matched with subjects having the same age, gender, residential area, and initial year in database, who did not attend the program. There were fewer traumatic injuries in the study group than in the control group. This difference was stronger in females. They concluded that the P.A.R.T.Y. program effectively reduced the incidence of traumatic injuries among its participants .^17^ In 2012 Ho and colleagues published a retrospective cohort study including 3659 juvenile justice offenders. In a before and after survey of 225 participants, a significant proportion of them stated that they were more receptive to modifying their risk-taking behavior. The incidence of subsequent traffic or violence-related offences was significantly lower for those who had attended the program compared to those who had not. They concluded that participating in an injury education program involving real-life trauma scenarios was associated with a reduced subsequent risk of committing violence or traffic-related offences, injuries, and death for juvenile justice offenders.^18^ On the whole, an injury prevention measure needs to address several topics. The abuse of drugs and alcohol as well as the use of cell-phones and the benefit of using a helmet need to be discussed and knowledge about these topics needs to be mediated.^19-22^

However, the impact of injury prevention measures on young road users has not been well understood and long-term results are missing. To positively influence the behavior of young road users and to guide injury prevention measures, it is mandatory to analyze sociodemographic factors (i.e., gender, age, educational level) and to examine drivers' behavior to influence the high number of mortality and morbidity in RTCs.^23^ It is known that gender has been considered in relation to risky driving behavior in young drivers and it has been found that, in terms of risky behavior in road traffic, men are more willing to take risks than women.^24,25^ Yagil et al. reported that the rate of men's involvement in fatal road collisions is twice as high as women's.^26^ Furthermore, age is another negative predictor of risky driving behavior. It has been well established by studies and databases from various countries that young novice drivers are more frequently involved in traffic collisions than drivers in other age groups.^1,6,23,7^ In addition to these factors, the level of education also plays an influential role. There are noticeable differences in the social and socioeconomic distribution of RTCs, as measured in terms of either mortality or morbidity.^27-29^ Recent reviews show that the bulk of evidence accumulated so far strongly suggests that the risk of being injured is highest among people in less privileged socioeconomic groups or living in less privileged areas. ^28-30^

In this study, the P.A.R.T.Y. program was used to evaluate the influence on different sociodemographic factors: gender, age, residential area and level of education. A pre-post study design was used to describe the results of the first 2 years of evaluation of the P.A.R.T.Y. program in a Level-1-Trauma hospital in Germany. The results of the presented study can be used to optimize the prevention measure. The program should target road traffic users with a high risk of injury. In addition, improvements can be done to the program, to better address road users that are less strong affected by the program so far. 

## Methods 

**Questionnaire development: **

The starting point for our questionnaire development draws from statistical data of the area of North Rhine Westphalia (2010) on young road users aged between 13 and 20 years who were injured and killed in RTCs. In a next step, we examined how these RTCs are related to the use of specific means of transportation. Most of the collisions occurred in car use, followed by bicycle, moped or motorcycle use and as pedestrians. Looking only at our target group of the 14 to 17-year-olds, the proportion of RTCs in the context of passengers in a car is significantly lower and the contexts of bicycle and motorbike use account for about 60% of this target group. Due to this, the risk-behavior and risk-awareness of pedestrians, cyclists and users of motorized two-wheelers was identified.

In the field of traffic safety research, the generic error model system (GEMS) is a frequently employed approach.^31^ The GEMS distinguishes two basic categories of risk behaviors - 'errors' and 'violations', each of which is determined by different psychological mechanisms and therefore requires different interventions. 'Errors' are unintentional deviations from safe practices and reflect inadequate capabilities (e.g., due to inexperience) or unfavorable temporary conditions (e.g., fatigue). 'Violations', on the other hand, are deliberate deviations from safe practices (e.g., consciously crossing a red traffic light) that reflect a person's behavioral motivation (e.g., the desire to save time). The GEMS is also used to analyze risk-behavior among young people. ^32,33^ In addition to the distinction of 'errors' and 'injuries', Elliot et al. introduced the distinction between three other types of risk indicators: 'dangerous play','lack of protective behavior' and 'unsafe crossing'. ^32^ Based on these theoretical considerations, a series of standardized questions for the recording of risk-behavior and risk-awareness of young road users, such as pedestrians, cyclists and motorized two-wheel users have been established and aided by the development of our questionnaire. 

**Questionnaire measures:**

The questionnaire contains three sections with different types of questions (in total 22 questions) to evaluate the students' interpretation of risk-behavior and risk-awareness depending on different sociodemographic factors.

**Section 1: Risk-behavior items**

The pre-questionnaire included ten questions: Have you driven your car/ motorbike after drinking alcohol or taking drugs? Have you been given a lift by someone who had consumed alcohol or taken drugs? Do you wear a seatbelt when driving a car? Do you wear a helmet when you drive a motorbike/ moped? Do you fasten your helmet's chinstrap when you drive a motorbike/ moped? Do you wear a helmet when you ride a bicycle? Do you wear a helmet when you go rollerblading or skateboarding? Do you make calls on your mobile phone when driving/ cycling? Do you often listen to music through headphones when driving/ cycling? Do you observe the speed limit? Participants could choose from the following responses based on a five-point scale: “always; frequently; sometimes; rarely or never”. When evaluating question one, answers were only considered for those students who had a driver's license.

**Section 2: Risk-awareness items**

The second section included seven questions. Which of the following situations would you consider to be high-risk/ dangerous? Jumping into a lake without knowing how deep the water is? Riding as a passenger in the boot of a car? Cycling without a helmet? Overtaking a car on a mountain road or a bend? Speeding, Skydiving, Drunk-driving? Participants could choose from the following responses: “strongly agree; somewhat agree; somewhat disagree or strongly disagree”. 

**Section 3: Belief/opinion items**

The third section included five questions about what students think about themselves in various situations and about how they would react in different situations. I generally consider myself to be a “safety-loving” person. I try to avoid dangerous/risky situations. I sometimes take a risk instead of weighing up a situation. In the past 30 days, I have done things that some people would consider to be dangerous. Having fun while also being aware of my health/safety and that of other people is important to me. Possible responses: “yes, completely true; somewhat true; not really true and no, not at all true”. Questionnaires were written in German language. The post-questionnaire was linguistically adapted and aimed at actions in the future.

**Data collection:**

All schools were selected by a random principle in the City of Cologne, Germany. Only students of classes 9 to 11 (age 14 to 17) were included, due to the fact that students between 14 and 17 years of age start driving cars (e.g. accompanying driving) and motorbikes and students under the age of 14 were too young to participate. Finally, comprehensive schools and secondary modern schools (comprehensive schools offer a higher educational level than secondary modern schools) were randomly selected to participate in the program. 

Before participating, a consent form was signed by the parents of the students. Medical staff was trained to help with data collection. A pre-questionnaire was explained and handed out by our medical staff after the students arrived at the hospital and before attending the program. The post-questionnaire was filled out at the end of the same day. Students who were absent on the day of data collection or students who did not have written consent from their parents to participate in the evaluation were excluded from the pre-and post-evaluation. We also removed results of driving-related items of participants who were too young to have a driver's license. The evaluation was carried out to see whether the responses to individual questions from each section showed statistical differences between the pre- and post-evaluation forms.

**Data analysis:**

A total of four sociodemographic factors (subgroups) were compared: gender (female versus male), age (≤ 15 years of age versus ≥16 years of age), residential area (≤ 20.000 residents versus ≥20.001 residents) and type of school (comprehensive school versus secondary modern school).

For data analysis, we did not present the result of each single question. We rather combined the results of each set of questions after testing all questions for reliability using Cronbach's alpha. We found Cronbach's alpha between 0.67 and 0.83 ( Table 1). A sufficiently good reliability is achieved when Cronbach's alpha is about 0.7. Therefore, we got one meaningful result for each section that allows comparison in each subgroup. 

**Table 1:** Cronbach's alpha for all three sections pre- and post-evaluation.

Sections	Cronbach's Alpha	Mean (±SD)	t-test
1st section pre	0.67	2.25 (0.67)	
1st section post	0.77	2.10 (0.68)	p=.006
2nd section pre	0.70	2.00 (0.50)	
2nd section post	0.73	1.87 (0.53)	p<.001
3rd section pre	0.82	2.32 (0.74)	
3rd section post	0.83	2.19 (0.76)	p=.004

Descriptive statistics were reported as means (±SD) for continuous measures and proportions (%) for categorical measures. We analyzed data using paired t-test and since the data are not normally distributed, the results of the t-tests were ensured by the Wilcoxon signed-rank test. Because this analysis was exploratory, we did not undertake any adjustment for tests that we conducted. All tests for significance were at the 5% significance level. Statistical analyses were performed with SPSS (Statistical Package for the Social Sciences Version 22). 

**Characteristics of the participants: **

A total of 193 students aged between 14-17 years (mean: 15.9) were included into our study. They attended the program between 2011 and 2013. 44% were male students. 49.7% live in a medium-sized or large town (≥ 20.000 residents). 56.5% are students of a comprehensive school. 

## Results

**Results by gender: **

Our data show significant differences in the pre-post analysis in both groups (male and female). Male students show changes regarding risk-behavior (Section 1: pre: 2.31, SD: 0.64 vs. post: 2.14, SD: 0.68; p=.008). In the female group, we found significant changes in all three sections. Female students show better results regarding risk-behavior and risk-awareness. (Section 1: pre: 2.21, SD: 0.70 vs. post: 2.08, SD: 0.68; p= .037; Section 2: pre: 1.88, SD: 0.44 vs. post: 1.73, SD: 0.54; p<.001). (Table 2)

**Table 2:** Results by gender.

	Male students				Female students			
	Pre (Mean/SD)	Post (Mean/SD)	t-test	Wilcoxon-test	Pre (Mean/SD)	Post (Mean/SD)	t-test	Wilcoxon-test
Items of the 1st section(1 = no risk-behavior; 5 = risk-behavior)	2.31 (0.64)	2.14 (0.68)	p=.008	p=.009	2.21 (0.70)	2.08 (0.68)	p=.083	p=.037
Items of the 2nd section(1= risk-taking activities; 4= no risk-taking activities)	2.21 (0.52)	2.13 (0.42)	p=.120	p=.206	1.88 (0.44)	1.73 (0.54)	p<.001	p<.001
Items of the 3rd section(1=not venturesome; 4=venturesome)	2.57 (0.74)	2.53 (0.73)	p=.513	p=.509	2.17 (0.71)	1.99 (0.70)	p=.002	p=.001

**Results by age:**

When separating all students in two age groups (≤ 15-year-old and ≥16-year-old students), we found significant changes in responding regarding all three sections in the group of the ≥ 16-year-old students (Section 1: pre: 2.30, SD: 0.66 vs. post: 2.08, SD: 0.65; p= .007; Section 2: pre: 2.02, SD: 0.48 vs. post: 1.91, SD: 0.54; p.001). The younger participants (≤ 15-year-old) only show significant changes in section 2 (risk-awareness). Interestingly, results of the pre-tests show that the younger students (≤ 15-year-old) respond a bit more in the direction of a correct behavior and assess some situations as even more risky than the older students do. (Table 3)

**Table 3:** Results by age.

	≤ 15 years				≥ 16 years			
	Pre (Mean/SD)	Post (Mean/SD)	t-test	Wilcoxon-test	Pre(Mean/SD)	Post (Mean/SD)	t-test	Wilcoxon-test
Items of the 1st section(1 = no risk-behavior; 5 = risk-behavior)	2.17 (0.70)	2.13 (0.72)	p=.469	p=.312	2.30 (0.66)	2.08 (0.65)	p=.007	p=.001
Items of the 2nd section(1= risk-taking activities; 4= no risk-taking activities)	1.97 (0.52)	1.81 (0.51)	p=.007	p=.005	2.02 (0.48)	1.91 (0.54)	p=.005	p=.001
Items of the 3rd section(1=not venturesome; 4=venturesome)	2.12 (0.66)	2.06 (0.70)	p=.467	p=.519	2.44 (0.77)	2.27 (0.78)	p=.001	p<001

**Results by residential area:**

In the group of the students of a village or small town, results show significant changes regarding risk-behavior and risk-awareness. Compared to students who live in medium-sized or large cities, we found significant changes regarding risk-awareness and in section 3. (Table 4) 

**Table 4:** Results by residence.

	village or small town				medium-sized or large city			
	Pre (Mean/SD)	Post (Mean/SD)	t-test	Wilcoxon-test	Pre(Mean/SD)	Post (Mean/SD)	t-test	Wilcoxon-test
Items of the 1st section(1 = no risk-behavior; 5 = risk-behavior)	2.21 (0.63)	2.08 (0.66)	p=.023	p=.011	2.29 (0.73)	2.13 (0.70)	p=.099	p=.049
Items of the 2nd section(1= risk-taking activities; 4= no risk-taking activities)	1.98 (0.51)	1.82 (0.54)	p=.001	p<.001	2.03 (0.48)	1.95 (0.51)	p=.051	p=.045
Items of the 3rd section(1=not venturesome; 4=venturesome)	2.23 (0.73)	2.14 (0.78)	p=.104	p=.031	2.42 (0.76)	2.26 (0.73)	p=.012	p=.015

**Results by level of education:**

For students of comprehensive schools, we found significant changes in all sections with better results after attending the program (Section 1: pre: 2.13, SD: 0.62 vs. post: 1.95, SD: 0.64; p= <.001; Section 2: pre: 1.96, SD: 0.49 vs. post: 1.75, SD: 0.48; p<.001). In the group of secondary modern school students, we only found significant changes in section 3. (Table 5)

**Table 5:** Results by level of education.

	Comprehensive school (high level of education)				Secondary-modern school (low level of education)			
	Pre (Mean/SD)	Post (Mean/SD)	t-test	Wilcoxon-test	Pre(Mean/SD)	Post (Mean/SD)	t-test	Wilcoxon-test
Items of the 1st section(1 = no risk-behavior; 5 = risk-behavior)	2.13 (0.62)	1.95 (0.64)	p=.001	p<.001	2.46 (0.73)	2.38 (0.66)	p=.519	p=.841
Items of the 2nd section(1= risk-taking activities; 4= no risk-taking activities)	1.96 (0.49)	1.75 (0.48)	p<.001	p<.001	2.08 (0.51)	2.09 (0.56)	p=.851	p=.722
Items of the 3rd section(1=not venturesome; 4=venturesome)	2.22 (0.73)	2.13 (0.75)	p=.054	p=.013	2.49 (0.75)	2.30 (0.77)	p=.030	p=.033

## Discussion

The present work analyzed the impact of an injury awareness program on young students regarding different sociodemographic factors. Measuring the impact of any kind of prevention measure is difficult, due to various unpredictable side effects. However, it is imperative to understand how a prevention measure works and to find out how several subgroups are influenced by the measure. It is known that male road users are at a higher risk than female road users for sustaining a road traffic injury and there is also an impact regarding the level of education.^34^

The presented data show differences in all subgroups (gender, age, residential area and type of school). Looking at the pre-test, we found females being slightly more aware of the right behavior in road traffic and of what kind of situations should be considered high risk/ dangerous. Furthermore, after attending the program they show better results compared to the male students. There is evidence that female road users more often show a protective behavior. They are less involved in alcohol-related crashes and speeding-related crashes than male road users.^35^ It is also known that male road users are at a higher risk to sustain more severe injuries by cars and other motor vehicles.^36^ The P.A.R.T.Y. program seems to have a greater impact on female students than it does on male students. As male road users represent a large risk group, it is important that preventive measures address this group in particular in order to reduce RTCs and their consequences.

While looking at different age groups, the younger group shows better results in the pre-tests, but comparing the pre- and post-questionnaires, the group of ≥16-year-old students show significant results in all three sections. The effect on the young group seems not as strong as the effect on the older group of students. Nevertheless, results of other studies show that in children, the right behavior regarding road safety (e.g. wearing helmets) decreases with increasing age. Children tend to wear helmets more often than adolescents. The rate of helmet use in children is higher if their parents also wear helmets.^37,38^ Even before attending the prevention measure, it seems that younger students are more aware of the right behavior and of certain risky situations. But we measured a stronger effect on the older group of students in the pre-post comparison. This is of interest since students do their driver's license for motorbikes at the age of 16 and count as a high-risk group in road traffic. 

During our evaluation, we focused on participants of two different school types. One group of students of a comprehensive school and one group of students of a secondary modern school. We found the P.A.R.T.Y. program to have a higher impact on students of a comprehensive school regarding both risk-awareness and risk-behavior. In our study, comprehensive school students are of a higher educational level compared to students of the secondary modern school. Multiple studies show a direct correlation between educational level and morbidity and mortality during RTCs. Low socioeconomic and educational level has been shown to increase the risk of fatal or non-fatal traffic injuries.^39-41^ Hence, it is important to reach students of low socioeconomic and educational levels in order to influence their understanding and knowledge of risk-behavior and risk-awareness. 

Data were also analyzed regarding different residential areas. Students from villages or small towns and others from medium-sized or large cities participated. We did not find strong differences regarding risk-behavior or risk-awareness between these two groups. 

The present pilot study has certain limitations as it relies on a simple pre-post design. It is a single center evaluation, as we only focused on one major trauma center and not on different centers across the country. No long-term results are available. However, a prospective and multicenter evaluation of the program might help to improve the setting of the program to better focus on the high-risk group of young road users. 

## Conclusion

Morbidity and mortality due to RTCs is a major problem in the group of young road users. Especially male road users between 14 and 17 years of age with a low educational level are at high risk to sustain road traffic injuries. We set up an injury prevention program to analyze the effect on risk-behavior and risk-awareness of different subgroups of young students. Our results show that the P.A.R.T.Y. program has a stronger effect on young female students compared to male students. Additionally, a significant effect was measured on students ≥16 years of age and on students with a higher educational level. Prevention measures need to be evaluated and further improved in order to address especially the high-risk group of young, male road users with a lower educational status. 

**Acknowledgements: **

The authors thank the participating staff members of Cologne-Merheim-Medical-Center for their collaboration.

TB contributed to study design, acquisition and interpretation of data, recording of paper and analyzing data. MK provided statistical advice on study design. PK, MC, BB and US conceived of the study, provided statistical advice on study design. PK, MC, BB and US contributed to analysis and interpretation of data and revision of the article. All authors read and approved the final manuscript for publication.
